# A Novel Butyrate Derivative, Zinc Dibutyroyllysinate, Blunts Microphthalmia-Associated Transcription Factor Expression and Up-Regulates Retinol and Differentiation Pathway mRNAs in a Full-Thickness Human Skin Model

**DOI:** 10.3390/ijms26062442

**Published:** 2025-03-09

**Authors:** William R. Swindell, Krzysztof Bojanowski, Geovani Quijas, Ratan K. Chaudhuri

**Affiliations:** 1 Department of Internal Medicine, University of Texas Southwestern Medical Center, Dallas, TX 75390, USA; 2 Sunny BioDiscovery Inc., Santa Paula, CA 93060, USA; kbojanowski@sunnybiodiscovery.com (K.B.); geovani@sunnybiodiscovery.com (G.Q.); 3 Sytheon (a Hallstar Company), Parsippany, NJ 07054, USA; ratan@sytheonltd.com

**Keywords:** dermatopharmacology, melanocyte, prolidase, retinol, RNA-seq, zinc salt

## Abstract

Lysine, butyric acid, and zinc play important roles in skin homeostasis, which involves aging, inflammation, and prevention of skin barrier disruption. This bioactivity spectrum is not replicated by any one topical compound currently in use. Our purpose in this study was to characterize a novel compound, zinc dibutyroyllysinate (ZDL), consisting of zinc with lysine and butyric acid moieties. We used RNA-seq to evaluate its effect on gene expression in a full-thickness skin model. We show that lysine alone has minimal effects on gene expression, whereas ZDL had greater transcriptional bioactivity. The effects of ZDL included an increased expression of genes promoting epidermal differentiation and retinol metabolism, along with a decreased expression of microphthalmia-associated transcription factor (*MITF*) and other melanogenesis genes. These effects were not replicated by an alternative salt compound (i.e., calcium dibutyroyllysinate). ZDL additionally led to a dose-dependent increase in skin fibroblast extracellular matrix proteins, including collagen I, collagen IV, and prolidase. Loss of melanin secretion was also seen in ZDL-treated melanocytes. These results provide an initial characterization of ZDL as a novel topical agent. Our findings support a rationale for the development of ZDL as a skincare ingredient, with potential applications for diverse conditions, involving melanocyte hyperactivity, pigmentation, inflammation, or aging.

## 1. Introduction

Acylation is essential for all life forms, and it is principally found in nucleic acids [1], proteins [2], and lipids [3,4]. Protein acetylation is a well-studied regulatory mechanism for cellular processes ranging from gene expression and DNA repair to metabolism [5]. It can affect protein function, stability, localization, and interactions with other molecules [6]. This posttranslational modification is typically added to lysine residues and is capable of triggering profound alterations [7]. For example, lysine acetylation in histones serves as an epigenetic hallmark capable of changing chromatin structure, and thus gene expression patterns [8].

Butyric acid is a short chain fatty acid with an impressive breadth of activity in diverse organ systems, including skin [9]. In skin, butyrate has anti-inflammatory activity and contributes to immune defense by modifying the cutaneous microbiome [10]. It also helps provide nourishment to skin cells and is protective against infection and UV radiation [11]. Prior work, for example, demonstrated the effectiveness of topical butyric acid against UVB-induced inflammation in murine skin, preventing skin ulceration and loss of epidermal thickness with repression of both IL-6 and IL-8 [12]. These immunomodulatory effects may be mediated by interactions with GPR43 that ultimately serve to repress the release of pro-inflammatory cytokines in response to skin injury [12].

Thus, topical butyrate administration may have therapeutic applications with a therapeutic potential on inflammatory skin diseases. Furthermore, butyrate can be effective not only in overt skin diseases, but also as an ingredient in preventive topical products. Unfortunately, the main practical limitation for use of butyrate in dermatology is its unfavorable sensorial (unpleasantly pungent odor) and physicochemical properties. Butyric acid esters are also unstable and promptly release butyric acid even at room temperature. In contrast, butyrylation of lysine is regarded as a potentially therapeutic product of acetylation between these two acids [13].

Dermatological products commonly incorporate zinc salts [14], and these formulations are well-tolerated without safety concerns [15]. Zinc has multifaceted cellular roles and serves as a cofactor for a broad range of biochemical reactions that impact tissues in numerous ways related to homeostasis, growth/development, and cellular differentiation [16]. As a consequence, zinc is viewed as an essential microelement with an important role in prevention of skin disease [17]. Topical formulations have previously incorporated zinc due to its antimicrobial activity and effectiveness against acne, providing a strategy to avoid adverse effects that may be associated with systemic treatments [18,19]. With regard to acne, zinc’s therapeutic efficacy may be related to its anti-inflammatory activity against the bacterium *Cutibacterium acnes* [20,21], although other important mechanisms may include the repression of lipase and 5α-reductase enzymes [22] and a decreased production of sebum [20]. Topical zinc formulations may also be used to combat body odor though the formation of zinc salts with short chain fatty acids [14]. Beyond this, zinc modulates the expression and metabolism of filaggrin (FLG), an important barrier protein critical for the retention of moisture in the cornified layer. For example, zinc can increase FLG production by up-regulating serine protease 8 (PRSS8) activity, while suppressing FLG metabolism via the inhibition of peptidyl arginine deiminase (PAD) activity [23,24].

Calcium is another versatile cation with a broad activity spectrum that includes a ubiquitous function as a second messenger within signaling cascades. The role of calcium as a driver of keratinocyte differentiation is well-established, and the establishment of calcium gradients within the epidermis contributes significantly to skin barrier formation [25]. More recent work has also started to uncover the regenerative effects of calcium that facilitate the maintenance of stem cell activity to promote a healthy skin phenotype [26]. These effects are partly related to the regulatory activity of calcium within intracellular signaling cascades that control stem cell fate and the balance between quiescence and mitotic activation [27].

**Figure 1:**
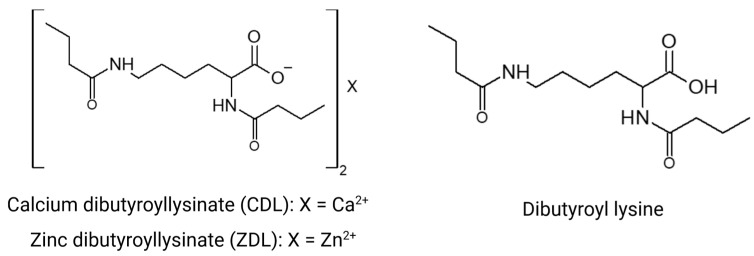
Chemical structures. The structure of compounds calcium dibutyroyllysinate (CDL) and zinc dibutyroyllysinate (ZDL). The intermediate of these two compounds is also shown (dibutyroyl lysine).

Based on the roles played by butyric acid, lysine acylation, zinc, and calcium metal ions in the skin, we have designed two novel lysine analogs, zinc dibutyroyllysinate (ZDL) and calcium dibutyroyllysinate (CDL), by selecting two types of ingredients to modify the structure of lysine-butyric acid and divalent metal ions Zn^2+^ and Ca^2+^ (Figure 1). The purpose of this study was to investigate the genomic and transcriptomic changes triggered by ZDL and CDL versus lysine using a three-dimensional human skin model (EpiDermFT^®^, MatTek Life Sciences, Ashland, MA, USA), with the ultimate goal of developing new bioactive ingredients that can be incorporated into topical applications.

## 2. Results

### 2.1. Differential Expression Analyses Overview

Differential expression testing was performed for three comparisons of interest (LYS vs. CTL, CDL vs. CTL, ZDL vs. CTL). The fewest differentially expressed genes were identified in the LYS (Lysine) vs. CTL comparison (Figure S1A–D). The LYS and CTL samples overlapped in the two-dimensional principal component space (Figure S1A) and the raw *p*-value distribution was uniform (Figure S1B). Overall, 18 differentially expressed genes (DEGs) were identified at a stringent significance threshold (FDR < 0.10 with FC > 1.50 or FC < 0.67), with 201 DEGs identified based upon a more permissive threshold (*p* < 0.05 with FC > 1.50 or FC < 0.67). The number of increased and decreased genes was similar (Figure S1C) and the average FC was stable between low- and high-expressed genes (Figure S1D).

A larger number of differentially expressed genes was identified in each of the other two comparisons (Figure S1E–L). ZDL samples were non-overlapping with CTL samples in two-dimensional principal component space (Figure S1I). In each comparison, raw *p*-value distributions were L-shaped, consistent with significant treatment effects (Figure S1F,J). The ZDL response was associated with more decreased than increased genes (Figure S1K). In contrast, the number of increased and decreased genes was similar with respect to CDL responses (Figure S1G). In each comparison, the average FC was similar between low- and high-expressed genes (Figure S1H,L).

**Figure 2:**
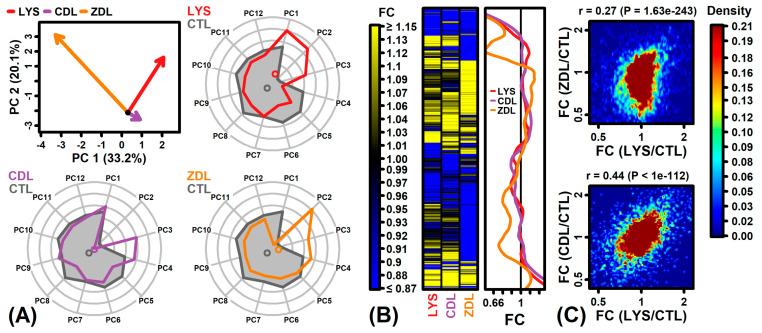
Differential expression overview. (**A**) Principal component (PC) scores. The upper left plot shows vectors connecting the CTL sample bivariate mean to the bivariate mean of treated samples (PC1 and PC2). Longer vectors correspond to stronger treatment effects, with arrows oriented in the same direction corresponding to more similar treatment effects. Radial plots show average scores for CTL and treated samples (PC1–PC12, *n* = 3 replicates per treatment). (**B**) Heatmap. The heatmap shows the FC estimates for a filtered set of 7234 genes with the strongest evidence for differential expression in any one of the three treatments. Genes are hierarchically clustered based on the Euclidean distance. Loess-fitted FC trends are also shown to the right of the heatmap. (**C**) Scatterplots. Scatterplots show the association between the FC estimates for each treatment and the LYS/CTL comparison. The Spearman rank correlation is shown with *p*-value (top margin). Colors correspond to gene density (see color scale).

The three compounds had differing effects on principal component (PC) scores (Figure 2A). LYS had a large effect on PC1 (33.2% of the gene expression variance) whereas ZDL had a more substantial impact on PC2 (20.1%) (Figure 2A). In contrast, CDL primarily impacted PC scores 3 and 4 (Figure 2A). A cluster analysis demonstrated a large fraction of genes uniquely down-regulated by ZDL (Figure 2B). Although the effects of CDL and ZDL were correlated with those of LYS, there was a weaker correlation between the effects of ZDL and LYS (Figure 2C).

### 2.2. LYS-Regulated Genes

**Figure 3:**
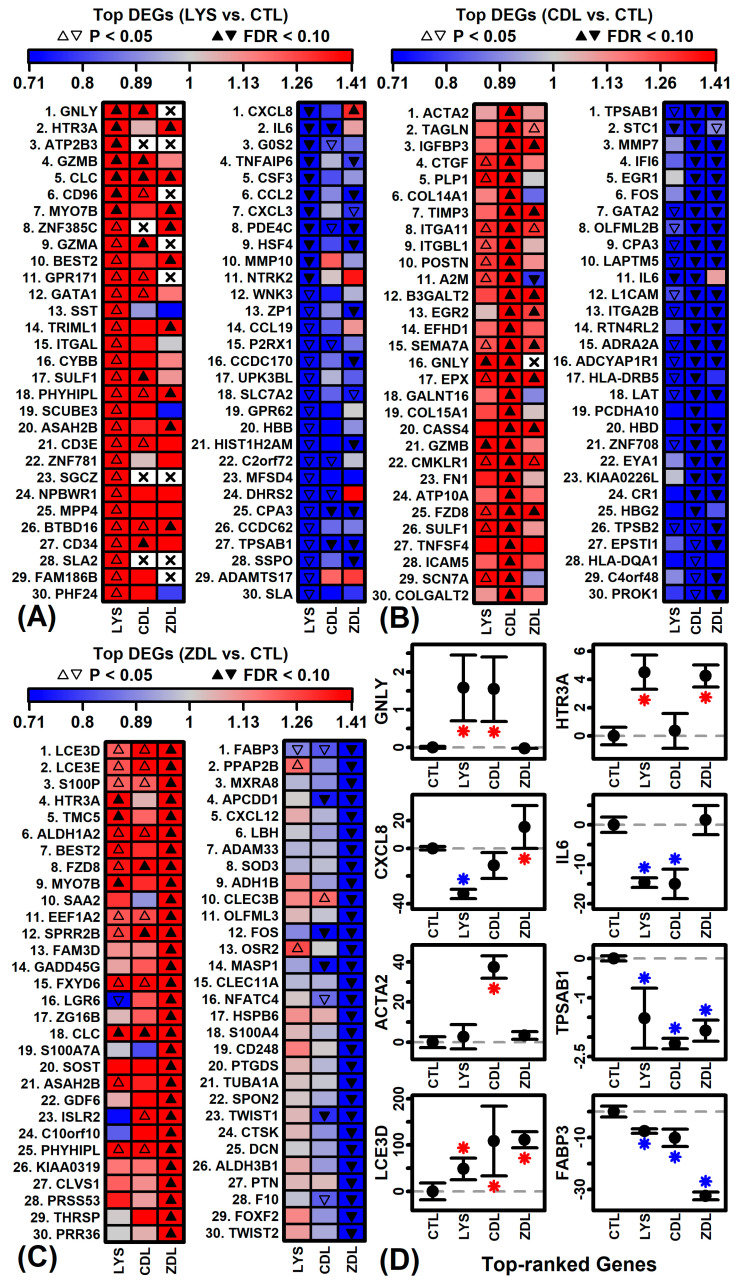
Top-ranked differentially expressed genes. (**A**) LYS vs. CTL. (**B**) CDL vs. CTL. (**C**) ZDL vs. CTL. In (**A**–**C**), the 30 top-ranked differentially expressed genes are shown (increased and decreased), with gene ranking determined based upon *p*-value. FC is indicated by color (red: increased; blue: decreased) and significance is indicated by symbols (open symbols, *p* < 0.05; filled symbols, FDR < 0.10). (**D**) Average expression per treatment for selected genes (*n* = 3 replicates per treatment). The average expression is shown for each treatment with error bars spanning ± 1 standard error. Expression is quantified based upon FPKM standardized such that the average expression in the CTL treatment is zero. Asterisk symbols indicate significant differences relative to the CTL treatment (*p* < 0.05; red: increased expression; blue: decreased expression).

**Figure 4:**
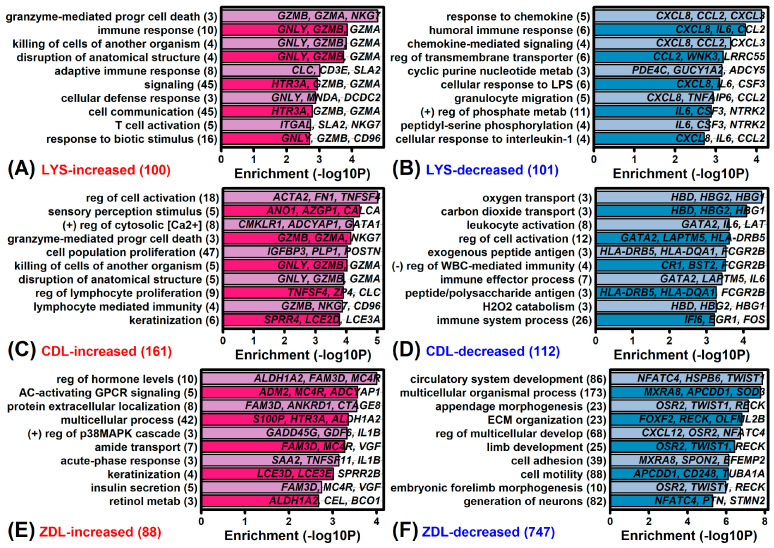
Gene Ontology (GO) Biological Process (BP) terms. Analyses were performed based upon genes differentially expressed in the (**A**,**B**) LYS vs. CTL, (**C**,**D**) CDL vs. CTL, and (**E**,**F**) ZDL vs. CTL comparisons. Differentially expressed genes in each analysis were identified based upon the same significance threshold (*p* < 0.05 with FC > 1.50 or FC < 0.67). The number of differentially expressed genes included in each analysis is indicated in parentheses (lower-left). Examples of differentially expressed genes associated with each GO BP term are listed within each figure.

Genes most strongly increased by LYS included granulysin (*GNLY*), 5-hydroxytryptamine receptor 3A (*HTR3A*), and ATPase plasma membrane Ca2+ transporting 3 (*ATP2B3*) (Figure 3A). Genes most strongly decreased by LYS included C-X-C motif chemokine ligand 8 (*CXCL8*), interleukin 6 (interferon, beta 2) (*IL6*), and G0/G1 switch 2 (*G0S2*). The expression of *GNLY* was up-regulated by LYS and CDL but was not well expressed in ZDL samples (Figure 3D). Expression of *CXCL8* was down-regulated by LYS but no other treatments (Figure 3D). As a group, LYS-increased genes were associated with granzyme-mediated programmed cell death, cytolysis, and immune response (Figure 4A). LYS-decreased genes were associated with the response to chemokine, humoral immune response, and cellular response to LPS (Figure 4B).

### 2.3. CDL-Regulated Genes

Genes most strongly up-regulated by CDL included actin alpha 2 smooth muscle (*ACTA2*), transgelin (*TAGLN*), and insulin-like growth factor binding protein 3 (*IGFBP3*) (Figure 3B). Genes most strongly down-regulated by CDL included tryptase alpha/beta 1 (*TPSAB1*), stanniocalcin 1 (*STC1*), and matrix metallopeptidase 7 (*MMP7*) (Figure 3B). Expression of *ACTA2* was not elevated by LYS or ZDL (Figure 3D). Expression of *TPSAB1* was uniformly repressed by all treatments but repressed most strongly by CDL (Figure 3D). As a group, CDL-increased genes were associated with the regulation of cell activation, sensory perception stimulus, and positive regulation of cytosolic calcium (Figure 4C). Likewise, CDL-decreased genes were associated with oxygen transport, carbon dioxide transport and leukocyte activation (Figure 4D).

### 2.4. ZDL-Regulated Genes

Genes most strongly increased by ZDL included late cornified envelope 3D (*LCE3D*), late cornified envelope 3E (*LCE3E*), and S100 calcium binding protein *p* (*S100P*) (Figure 3C). Genes most strongly decreased by ZDL included fatty acid binding protein 3 (*FABP3*), phospholipid phosphatase 3 (*PPAP2B*), and parvin alpha (*MXRA2*) (Figure 3C). The expression of *LCE3D* was uniformly elevated by all treatments (Figure 3D). Expression of *FABP3* was repressed by all treatments (Figure 3C). As a group, ZDL-increased genes were associated with the regulation of hormone levels, AC-activating GPCR signaling and protein extracellular localization (Figure 4E). Likewise, ZDL-decreased genes were associated with circulatory system development, appendage morphogenesis, and ECM organization (Figure 4F).

### 2.5. Retinol Metabolizing Genes

ZDL-increased genes were disproportionately associated with retinol metabolism (GO:0042572). Examples of such ZDL-increased genes include aldehyde dehydrogenase 1 family member A2 (*ALDH1A2*), carboxyl ester lipase (*CEL*), and beta-carotene oxygenase 1 (*BCO1*) (Figure 4E). *ALDH1A2* was noted to have the most significant increase, being among the top-ranked differentially expressed genes in the ZDL vs. CTL comparison (Figure 3C). Enrichment of this Gene Ontology term was not similarly observed with respect to LYS- or CDL-regulated genes (Figure 4A–D).

### 2.6. Differentiation-Associated Genes

**Figure 5:**
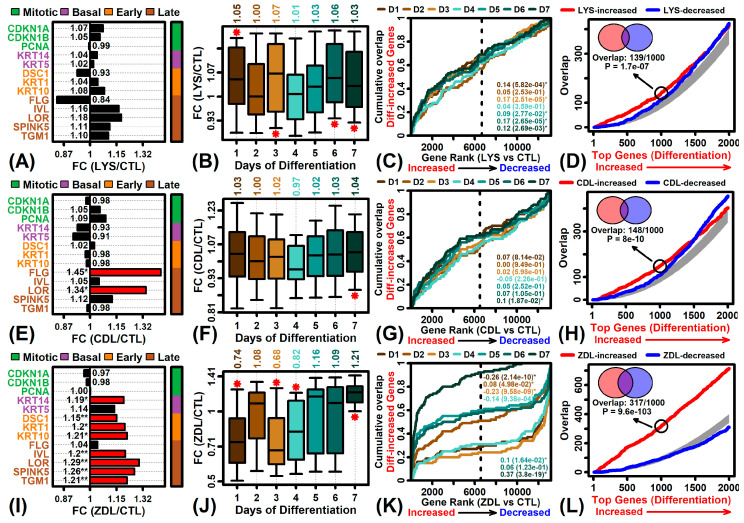
Differentiation-associated genes. (**A**,**E**,**I**) Marker genes associated with mitosis, basal layer KCs, early KC differentiation and late KC differentiation. The FC is listed within each figure (* *p* < 0.05, ** FDR < 0.10, *n* = 3 replicates per treatment) and red bars denote significant expression changes (*p* < 0.05). (**B**,**F**,**J**) Genes increased during epidermal regeneration (GSE52651). The top 50 genes up-regulated on each day of regeneration were identified (i.e., lowest *p*-values with FC > 1.0). Boxes outline the FC estimates for the middle 50% of these genes (whiskers: 10th to 90th percentiles; * *p* < 0.05). The average FC is listed in the top margin. (**C**,**G**,**K**) GSEA analysis of genes increased during epidermal regeneration (GSE52651). Plots show a cumulative overlap between the top 50 genes increased on each day of epidermal regeneration (vertical axis) and a list of genes ranked according to test compounds (horizontal axis). The area between each curve and the diagonal is shown with the *p*-value (area > 0: differentiation-increased genes enriched among test compound-increased genes; area < 0: differentiation-increased genes enriched among test compound-decreased genes). (**D**,**H**,**L**) Overlap with genes increased during epidermal regeneration (GSE52651). Genes were ranked based on the slope of expression increase over the 7-day time course (horizontal axis) and overlap at each rank was evaluated with respect to the same number of genes increased (red) or decreased (blue) by each test compound (vertical axis). The circle denotes the overlap between the top 1000 differentiation-increased and top 1000 test compound-increased genes, with corresponding Venn diagram, overlap, and *p*-value shown (upper left, Fisher’s exact test).

Test compounds increased the expression of genes associated with keratinization or development (Figure 4C,E,F). Consistent with this, CDL and ZDL significantly increased the expression of some or all late differentiation marker genes, such as *FLG*, *IVL* and *LOR* (Figure 5E,I; *p* < 0.05). ZDL increased the expression of early differentiation marker genes as well (e.g., *DSC1*, *KRT1*, *KRT10*; Figure 5I, *p* < 0.05). In contrast, LYS did not significantly alter expression of differentiation marker genes (Figure 5A).

Afterwards, we compared the effects of each compound to gene expression changes seen in neonatal human KCs placed on devitalized human dermis to generate a fully stratified and differentiated epithelium (GSE52651) [28]. There was limited but weak correspondence between the genes altered by LYS or CDL and those altered by differentiation (Figure 5B–D,F–H). However, there was a robust trend in which genes increased during late differentiation were up-regulated by ZDL (Figure 5J–L). Of the 50 genes most strongly up-regulated on day 7 of differentiation (GSE52651), more than 90% were increased by ZDL (i.e., FC > 1.0; Figure 5J). Conversely, genes up-regulated on day 1 of the time course were down-regulated by 26% on average with ZDL treatment (Figure 5J). Consistent trends were seen with GSEA analyses, i.e., genes increased on day 7 were enriched among ZDL-increased genes (Figure 5K, *p* = 3.8 × 10^−19^), whereas genes increased on day 1 were enriched among ZDL-decreased genes (Figure 5K, *p* = 2.14 × 10^−10^). Of the 1000 genes increased most strongly during the differentiation time course, 317 were also included among the 1000 genes most strongly increased by ZDL (Figure 5L, *p* = 9.6 × 10^−103^, Fisher’s exact test).

### 2.7. Aging-And Senescence-Related Genes

We next evaluated the expression of genes associated with aging and cellular senescence. Genes related to aging were identified based upon five database sources (NHGRI-EBI GWAS Catalog, Gene Ontology, Kyoto Encyclopedia of Genes and Genomes, GenAge, Aging Atlas) [29,30,31,32,33]. This allowed us to identify 54 skin-expressed genes associated with aging by at least three of the five database sources. On average, these 54 genes were not disproportionately increased or decreased by any of the three treatments (Figure S2A). However, there were several aging-related genes for which expression was altered by one or more of the treatments. The expression of superoxide dismutase 2 (SOD2), for example, was significantly decreased by all three treatments (Figure S2D). On the other hand, expression of PPARG coactivator 1 alpha (*PPARGC1A*) was significantly decreased by ZDL, but not by any other treatment (Figure S2D).

Genes related to cellular senescence, including pro-senescence and anti-senescence genes, were identified from the CellAge database [34]. There was no overall trend to suggest that the treatments disproportionately increased or decreased the expression of pro-senescence genes (Figure S2B); however, there were several pro-senescence genes uniquely down-regulated by ZDL, including inhibitor of growth family member 2 (*ING2*), AXL receptor tyrosine kinase (*AXL*), G protein subunit gamma 11 (*GNG11*), suppressor of cytokine signaling 1 (*SOCS1*), XIAP associated factor 1 (*XAF1*), insulin like growth factor binding protein 5 (*IGFBP5*), and angiotensinogen (*AGT*) (Figure S2E). Likewise, there was no overall trend to suggest that anti-senescence genes were disproportionately increased or decreased by LYS, CDL or ZDL (Figure S2C). However, we did identify some anti-senescence genes uniquely up-regulated by ZDL, including mago homolog exon junction complex subunit (*MAGOH*), casein kinase 2 alpha 1 polypeptide (*CSNK2A1*), SWI/SNF related BAF chromatin remodeling complex subunit ATPase 4 (*SMARCA4*) and glucose-6-phosphate dehydrogenase (*G6PD*) (Figure S2F).

### 2.8. MITF Expression and Melanoma-Dysregulated Genes

**Figure 6:**
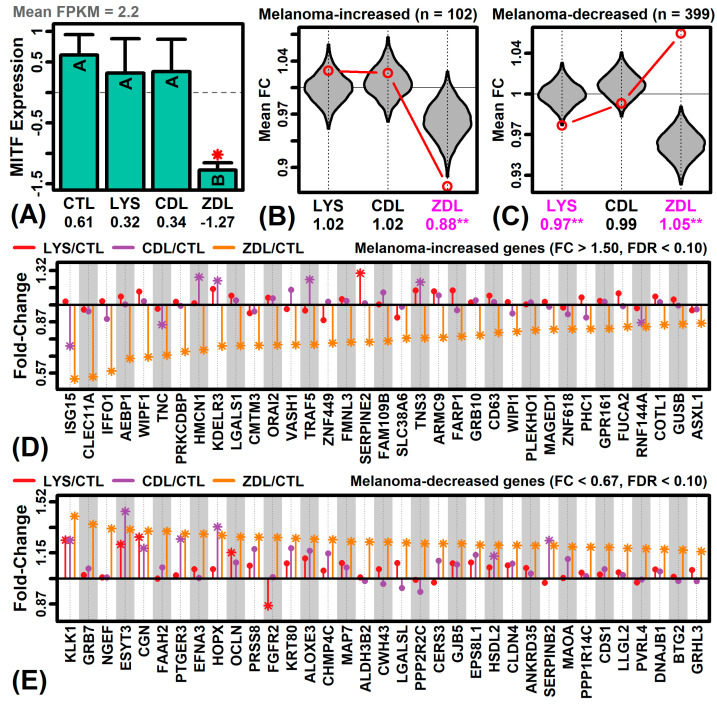
MITF expression and melanoma-dysregulated genes (GSE7553). (**A**) MITF expression. Z-scores were calculated based on FPKM values and the average value is shown for each treatment (±1 standard error, *n* = 3 replicates per treatment). Treatments without the same letter differ significantly (*p* < 0.05, Fisher’s LSD). A red asterisk (*) is used to denote treatments differing significantly from the CTL group. (**B**) Mean FC of melanoma-increased genes (FC > 1.50, FDR < 0.10, *n* = 3 replicates per treatment). (**C**) Mean FC of melanoma-decreased genes (FC < 0.67, FDR < 0.10). In (**B**,**C**), melanoma dysregulated genes were identified based on a comparison between primary melanoma skin lesions (*n* = 14) and normal human skin (*n* = 4) (GSE7553). Gray regions outline the null distribution obtained by randomly sampling genes and calculating the mean FC within each sample. The red circle denotes the observed mean FC and this value is also listed in the bottom margin (** *p* < 0.01; *p*-values calculated based on null distribution shown). (**D**) Melanoma-increased genes most strongly down-regulated by ZDL. (**E**) Melanoma-decreased genes most strongly up-regulated by ZDL. In (**D**,**E**), the FC estimate is plotted for each gene and treatment (*n* = 3 replicates per treatment), with FC calculated based upon comparison to the CTL group (asterisk: *p* < 0.05, circle: *p* > 0.05).

Microphthalmia-associated transcription factor (*MITF*) is repressed by HDAC inhibitor drugs in multiple cell types [35] and its expression is elevated in melanoma skin samples, wherein its activity may play a role in pathogenesis and treatment resistance [36]. Expression of *MITF* was uniquely suppressed by the ZDL treatment (FC = 0.63, *p* = 1.33 × 10^−8^, FDR = 7.65 × 10^−7^) but was not significantly altered by LYS or CDL (see Figure 6A). We further evaluated the effects of each compound on melanoma-dysregulated genes identified from a previously generated microarray dataset (GSE7553) [37]. This showed that melanoma-increased genes were, on average, decreased by ZDL (12%) but not by other compounds (Figure 6B). Conversely, melanoma-decreased genes were uniquely elevated by ZDL (5% on average) but not by other compounds (Figure 6C). Consistent with these findings, there was a genome-wide negative correlation (*r*_s_ = −0.267) between the effects of ZDL and expression shifts in melanoma skin (as compared to normal skin) (Figure S3C). This negative correlation, however, was not seen with respect to LYS or CDL (Figure S3A,B). We identified dozens of melanoma-increased genes uniquely down-regulated by ZDL, including C-type lectin domain containing 11A (*CLEC11A*), intermediate filament family orphan 1 (*IFFO1*), and AE binding protein 1 (*AEBP1*) (Figure 6D). We further identified several melanoma-decreased genes uniquely up-regulated by ZDL, including growth factor receptor bound protein 7 (*GRB7*), neuronal guanine nucleotide exchange factor (*NGEF*), fatty acid amide hydrolase 2 (*FAAH2*), and ephrin A3 (*EFNA3*) (Figure 6E).

### 2.9. RT-PCR Analysis

**Figure 7:**
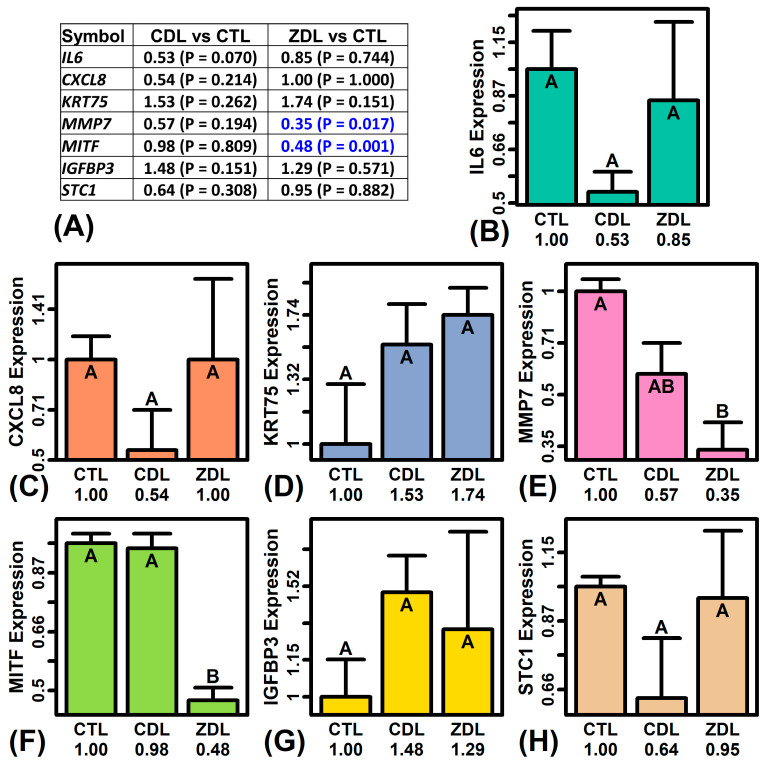
RT-PCR results. (**A**) FC estimates with *p*-values. *p*-values are shown in parentheses for each comparison to the CTL treatment (blue: FC < 1.00, *p* < 0.05). *p*-values were calculated using a two-sample two-sided *t*-test. (**B**–**H**) Average relative expression. The average relative expression of each gene (±1 standard error) is shown for each treatment (*n* = 2–3 replicates per treatment). Expression is normalized to the average value of the CTL treatment (arbitrarily scaled to one). Treatments not sharing the same letter differ significantly (*p* < 0.05, Fisher’s Least Significant Difference). In each analysis, relative expression was estimated using glyceraldehyde-3-phosphate dehydrogenase (*GAPDH*) as a reference gene.

In order to confirm the RNA-seq data, RT-PCR was used to evaluate the expression of seven genes with significant expression differences in the RNA-seq study (*IL6*, *CXCL8*, *KRT75*, *MMP7*, *MITF*, *IGFBP3*, *STC1*). RT-PCR was performed using a subset of the RNA samples that had been used in the RNA-seq study (*n* = 2–3 replicates per treatment). Consistent with RNA-seq findings, the expression of *MMP7* was repressed by ZDL to a lesser degree than by CDL (Figure 7A,E), whereas the expression of *MITF* was uniquely repressed by ZDL (Figure 7A,F).

### 2.10. ZDL Increases Abundance of Collagen I, Collagen IV and Prolidase but Decreases Melanin Secretion

**Figure 8:**
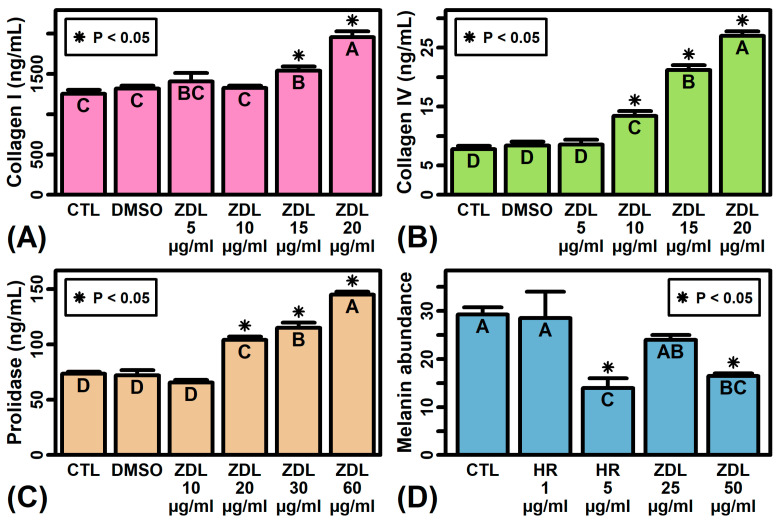
ZDL decreases melanin production but increases abundance of collagen I, collagen IV and prolidase. The mean is shown for each treatment ± 1 standard error (*n* = 2–3 replicates per treatment). Treatments without the same letter differ significantly (*p* < 0.05, Fisher’s least significant difference). Results are shown from untreated cells (CTL), cells treated with vehicle solution (dimethyl sulfoxide, DMSO), hexylresorcinol (HR), or different concentrations of ZDL. An asterisk (*) is shown of those treatments differing significantly from the CTL group (*p* < 0.05, Fisher’s least significant difference). Experiments in (**A**–**C**) were performed using fibroblasts whereas experiments in (**D**) were performed using B16 melanocytes.

Gene expression analyses demonstrated significant effects of ZDL on the expression of mRNAs associated with extracellular matrix and melanogenesis (e.g., *MMP7*, *MITF*). To further characterize this activity, we evaluated the effects of ZDL on relevant proteins in fibroblast and melanocyte cultures. Treatment of cultured human dermal fibroblasts with ZDL for 48 h led to a dose-dependent increase in collagen I abundance in cell culture media, with significantly increased collagen I levels observed in cells treated with ZDL at a concentration of 15–20 mcg/mL (Figure 8A). Likewise, we observed a dose-dependent increase in collagen IV production in ZDL-treated fibroblasts (Figure 8B). Treatment of fibroblasts with ZDL elicited a dose-dependent increase in the enzyme prolidase (Figure 8C), which plays an important role in collagen turnover and recycling. Finally, ZDL significantly decreased the melanin content of conditioned media from B16 melanocytes, with loss of melanin secretion similar to that seen in cells treated with the tyrosinase inhibitor hexylresorcinol (Figure 8D).

## 3. Discussion

This study compared the effects of zinc dibutyroyllysinate (ZDL) and calcium dibutyroyllysinate (CDL) as derivatives of butyrate against lysine in a three-dimensional human skin model (EpiDermFT^®^, MatTek Life Sciences, Ashland, MA, USA). These novel compounds (Figure 1) have not previously been evaluated, and this study thus provides a first analysis of their functional spectrum and potential utility in topical applications. Using high-throughput genomics and bioinformatic analyses, we show that LYS itself has limited transcriptional bioactivity, eliciting few transcriptional changes reaching statistical significance. CDL had a stronger transcriptional impact overall, with down-regulation of functionally significant genes such as *TPSAB1*, *STC1*, and *MMP7*. However, it was ZDL that triggered the most significant gene expression differences in skin equivalents. Clusters of genes uniquely modulated by ZDL were associated with the induction of late differentiation, inhibition of pigmentation, senescence and melanogenesis, as well as an increase in the expression of some retinol metabolizing genes. We further show that ZDL bolstered in vitro production of extracellular matrix proteins in skin fibroblasts (collagen I, collagen IV, prolinase) and was moreover able to repress melanin secretion in cultured melanocytes (Figure 8). These results lay groundwork for further studies of ZDL as a potential ingredient in skincare products designed to treat hyperpigmentation [38] or prevent the degradation of extracellular matrix that may occur due to intrinsic or extrinsic skin aging [39].

The process of keratinocyte differentiation involves finely tuned mechanisms needed to ensure effective barrier formation and maintenance [40]. While CDL and LYS only slightly elevated differentiation-associated genes, ZDL had the strongest trend, up-regulating > 90% of the genes associated with late epidermal differentiation (GSE52651) [28]. This activity may have significance in multiple skin conditions associated with skin barrier dysregulation, such as inflammatory skin diseases, genetic disorders, exposure to environmental insults, wounding, and aging [41]. In our study, ZDL up-regulated genes included the major players in barrier protection coding for late cornified envelope proteins (*LCE3E*, *LCE3D*), *FLG*, *IVL* and *LOR* [42,43,44]. Interestingly, ZDL up-regulated small protein rich proteins (SPRRs) [45], such as *SPRR2B*, which is located within the epidermal differentiation complex and encodes a cornified envelope (CE) protein participating in transglutaminase-mediated protein cross-bridging, thus playing a structural role in all stages of CE formation [46,47]. Further immunohistochemistry studies are needed to understand the effects of ZDL on the in situ abundance and distribution of these proteins throughout the epidermal layers. Our current findings, however, demonstrate the effects of ZDL on functionally significant mRNAs encoding differentiation proteins that are essential for epidermal stratification and barrier formation.

ZDL also uniquely repressed the expression of the microphthalmia-associated/melanocyte-inducing transcription factor (*MITF*) (Figure 6A). This protein regulates melanocyte development and is responsible for the generation of the proteins involved in pigmentation [48]. MITF is also a transcriptional master regulator of an array of genes implicated in melanoma [49,50]. Along these lines, ZDL tended to decrease the expression of the genes up-regulated in human melanoma skin samples (Figure 6B), and conversely increased the expression of the genes down-regulated in melanoma samples (Figure 6C). Our expression profiling studies were performed using three-dimensional skin cultures that do not include melanocytes, although *MITF* had detectable expression in all samples, suggesting that *MITF* is expressed by multiple skin cell types. Further studies to evaluate the effect of ZDL on *MITF* expression in melanoma cell lines will thus be an important component of future work, although it is notable that direct treatment of cultured melanocytes with ZDL repressed melanin secretion (Figure 8D). Our findings thus provide evidence to support further studies into the efficacy of ZDL as a depigmentation agent [51], for example in the setting of treatment-resistant vitiligo [38,52], or as a topical treatment for early-stage melanocytic lesions with potential for progression to melanoma [53].

ZDL was found to increase the expression of genes associated with retinol metabolism (Figure 4E). For example, ZDL uniquely up-regulated the expression of aldehyde dehydrogenase 1 family member A2 (*ALDH1A2*/*RALDH2*)—the enzyme catalyzing formation of retinoic acid from retinaldehyde (retinal) [54], which is interconvertible with retinol. In the skin, this activity may translate into increasing the pool of retinoic acid, which is the bioactive metabolite of retinol and a key homeostatic regulator in the skin utilized for anti-aging regimens [55,56]. The expression of *ALDH1A2* also decreases with age [57], and its up-regulation by ZDL appears to be part of a broader tendency of ZDL to counter many of the senescence-associated changes seen with replicative aging in skin fibroblasts (Figure S2). ZDL also had other effects that could potentially attenuate age-associated extracellular matrix degradation, such as eliciting an increased abundance of collagen I, collagen IV and prolidase in cultured fibroblasts (Figure 8). Prolidase activity, for example, synergistically interacts with extracellular type I collagen to help maintain the extracellular matrix [58,59]. Loss of collagen I and prolidase activity with aging may thus contribute to age-associated ECM degradation [58]. In this study, however, ZDL was effective at increasing both collagen I and prolidase levels, which could have preventative or rejuvenating effects within the dermal layer [60].

Together, our results suggest that the zinc salt of dibutyroyl lysine (zinc dibutyroyllysinate) may alter the expression of therapeutically important genes in human skin. Although the mechanism of this interference was not investigated here, it is tempting to hypothesize that it may involve epigenetic mechanisms [61], such as the regulation of histone acetylation [62,63]. While the precise mechanism of action of ZDL is currently under investigation in our laboratory, the present report demonstrates that topically applied ZDL has properties that may support overall skin homeostasis, assist with retinol metabolism, and participate in regulation of melanogenesis pathways.

## 4. Materials and Methods

### 4.1. Synthesis of Dibutyroyl Lysine

In tetrahydrofuran and distilled water, L-Lysine HCl and butyric anhydride were added and stirred for 12 to 16 h at 30 ± 5 °C. Sodium carbonate was then added to the reaction mixture and stirred for another 12 to 16 h at 30 ± 5 °C. Sodium chloride and hydrochloric acid were added and stirred for 30 min and the aqueous layer was separated. The organic layer was subjected to distillation under vacuum at <40 °C. The crude product was further purified by column chromatography. The purified product was characterized as dibutyroyl lysine using spectroscopic methods. Alternately, this compound can be made by reacting lysine with butyryl chloride. This is used as an intermediate and not available commercially.

### 4.2. Synthesis of Zinc Dibutyroyllysinate

Dibutyroyl lysine (2 moles) was mixed with methanol and zinc hydroxide (1.1 mole) and heated to 65 °C and maintained for 12 h. The reaction mixture was filtered to remove any undissolved salt. The filtrate was subjected to distillation under vacuum at <40 °C. The crude product was washed with acetone and n-heptane. The purified product was dried under vacuum at <40 °C and identified by 1H, 13C and mass spectral analysis. Alternately, this compound can be made by a reaction of dibutyroyl lysinate with ZnO (Fisher Scientific, Waltham, MA, USA). Zinc dibutyroyllysinate (INCI name) (Figure 1), also referred to as Bis (N2, N6-dibutyryl-lysine) zinc salt (2:1)] (CAS Index name) is available commercially (Sytheon, Parsippany, NJ, USA).

### 4.3. Synthesis of Calcium Dibutyroyllysinate

Dibutyroyl lysine was mixed with methanol and calcium hydroxide and heated to 65 °C and maintained for 12 h. The reaction mixture was filtered to remove any undissolved salt. The filtrate was subjected to distillation under vacuum at <40 °C. The crude product was washed with acetone and n-heptane. The purified product was dried under vacuum at <40 °C and identified by 1H, 13C and mass spectral analysis. Calcium dibutyroyllysinate (INCI name) (structure 2), also referred to as Bis (N2, N6-dibutyryl-lysine) calcium salt (2:1)] (CAS Index name) is available commercially (Sytheon, Parsippany, USA).

### 4.4. Sample Information

Gene expression profiling experiments were carried out using EpiDermFT tissues (MatTek Life Sciences, Ashland, MA, USA; cat no. EFT-400). EpiDermFT tissue cultures used in this study were generated by a single donor based upon technical documentation provided by the manufacturer (MatTek). Experiments included 4 treatments, including control (CTL), lysine (LYS), zinc dibutyroyllysinate (ZDL) and calcium dibutyroyllysinate (CDL) (*n* = 3 replicates per treatment). Test materials were dissolved in DMSO diluted with distilled water to a final concentration of 100 µg/mL. Test materials were then added to the apical surface of EpiDermFT tissues (3 µL/cm^2^) for 24 h. After the treatment period, tissues were rinsed and RNA extraction was performed using the commercial RNeasy Plus Mini kit (Qiagen, Germantown, MD, USA; cat. no. 74134). The QiaCube Connect robotic station was utilized to ensure a standardized extraction process. Total RNA was quality-assessed using the NanoDrop Lite (Thermo Fisher Scientific, Waltham, MA, USA).

### 4.5. RNA Sequencing

Sequencing data were generated by Azenta Life Sciences (South Plainfield, NJ, USA) on the Illumina HiSeq (2 × 150 bp) platform. Analyses were performed using raw FastQ files. Quality control reports from FastQ files were generated using FastQC [64]. TrimGalore was used for adaptor removal and quality trimming of reads [65]. Reads corresponding to rRNA were filtered out using bbmap [66]. The set of filtered and quality-trimmed reads were mapped to the hg38 genome using tophat2 [67]. Read counts for hg38 genes were generated using htseq-count [68] with FPKM values estimated using cufflinks [69]. Post-mapping quality control statistics were calculated using RSeQC [70] and RNA-SeQC [71].

An average of 46.9 million paired reads per sample were generated (range: 40.8–56.2 million) (Figure S4A). After the filtering steps, there remained an average of 46.1 million reads per sample (range: 40.1–55.4 million) (Figure S4B). An average of 98.3% of reads were successfully mapped to the UCSC hg38 genome sequence, with no less than 98.0% of reads mapped for any one sample (Figure S4C). An average of 99.6% of reads mapped to intragenic regions (Figure S4D), with 95.7% of reads assigned to annotated exons (Figure S4E). Only 0.11% of reads were mapped to rRNA sequence and among all samples the rRNA mapping rate was no greater than 0.12% (Figure S4F).

Differential expression testing was performed using edgeR (comparisons: LYS vs. CTL, CDL vs. CTL, ZDL vs. CTL) [72]. Differential expression testing was performed only for protein-coding genes having detectable expression in at least 2 of the 6 samples associated with a given comparison (LYS vs. CTL: 14,608 genes; CDL vs. CTL: 14,651 genes; ZDL vs. CTL: 14,578 genes). A gene was considered to have detectable expression if it was associated with at least one mapped read and if the lower limit of the FPKM 95% confidence interval was greater than zero. To control the false discovery rate (FDR), raw *p*-values from differential expression analyses were adjusted using the Benjamini–Hochberg method [73].

### 4.6. RT-PCR Analysis

cDNA was prepared using the High-Capacity RNA-to-cDNA™ Kit (Thermo Fisher Scientific, Richardson, TX, USA). Real-time quantitative PCR was performed using the BioRad iCycler iQ Detection System with AzuraView GreenFast qPCR Blue Mix LR (Azura Genomics, Raynham, MA, USA). PCR primers were purchased from Realtimeprimers (Elkins Park, PA, USA). Expression was evaluated based on cycle thresholds, defined as the number of replication cycles required for the fluorescent assay signal to exceed the background level [74]. Relative gene expression was quantified using the ΔΔCt method [75] using expression of glyceraldehyde-3-phosphate dehydrogenase (*GAPDH*) as a reference gene.

### 4.7. Collagen and Prolidase Production in Dermal Fibroblasts

Human neonatal fibroblasts were cultured in 48-well plates using Fibroblast Growth Media (FGM) and incubated overnight at 37 °C. Experiments were performed in triplicate. Cells were grown to confluence with media exchanged every 48 to 72 h. Confluent cells were washed and then treated with ZDL dissolved in FGM and 1.5% FBS for 48 h. Control cells were treated only with DMEM with 1.5% FBS for 48 h. The abundance of target proteins (collagen I, collagen IV, prolidase) was estimated based upon standard curves as described below.

Collagen I abundance was quantified based upon type I C-peptide standards with concentrations ranging from 0 to 640 ng/mL. Peroxidase-labeled anti procollagen type I-C peptide antibody (100 µL) was added to each well of an ELISA microplate along with 20 µL of sample or standard. The microplate was covered and incubated for 3 h at 37 °C. Wells were aspirated and washed followed by the addition of peroxidase substrate solution (100 µL). The plate was incubated for 15 min at room temperature, stop solution was added (100 µL of 1 N sulfuric acid), and the plate was then read using a microplate reader (450 nm).

Collagen IV standards were prepared and then either the standard or sample was added to type IV collagen plates and the reaction was allowed to proceed for 1 h (37 °C). Plates were washed and detection antibody solution was added (100 µL). The reaction was incubated for 1 h (37 °C). Plates were then re-washed and HRP conjugate solution (100 µL) was added and incubated for 30 min (37 °C). Plates were then re-washed and the color generation reaction was promoted by addition of substrate solution (100 µL) followed by incubation at room temperature for 10–30 min. Plates were then read using a microplate reader (460 nm) after the addition of stop solution (100 µL).

Prolidase standards were prepared and either the sample or standard (50 µL) was combined with HRP conjugated anti-prolidase antibody in a prolidase ELISA plate. Following a 1 h incubation period (37 °C), the plates were washed, and the color generation reaction was promoted by the addition of substrate solution (100 µL). Plates were then read using a microplate reader (460 nm) following the addition of stop solution (50 µL).

### 4.8. Melanin Secretion Assays

Test materials were stored at room temperature and stock solutions were prepared at 20 mg/mL in DMSO. Further dilutions were made in sterile distilled water immediately before adding to cells. B16-F1 melanocytes (cat. no. CRL-6323, ATCC, Manassas, VA, USA) were plated in Melanocyte Growth Medium [MGM, cat. no. 2201, lot no. 36237, ScienCell, Carlsbad, CA with Supplements cat. nos. 2252, 0503 (penicillin/streptomycin) and 0002 (FBS)] in a six well plate, in duplicates. The next day, cultures were observed through the Nikon Eclipse TS100 inverted microscope and determined to be at about 25% confluence. Cell growth medium was further supplemented with 3.7 mM tyrosine [76] and 100 nM alpha-MSH [77] to stimulate melanin production, and the test materials were added. We used the non-cytotoxic doses of hexylresorcinol and ZDL (1 µg/mL and 25 µg/mL, respectively) based on previously conducted cytotoxicity assays. We also tested higher doses for each test material (5 µg/mL and 50 µg/mL, respectively). The culture was maintained for seven days with medium changes every two days. Microscopic observation did not demonstrate any obvious signs of cytotoxicity, as defined by cell rounding and/or detachment. At the end of the experiment, cells were observed through the microscope, followed by trypsinization, pelleting and dissolution in the solvable reagent (Perkin Elmer, Waltham, MA, USA). Colorimetric signal acquisition was performed at 405 nm. The cell culture conditioned medium was analyzed for pigmentation at the same wavelength with the SpectraMax i3x Multi-Mode Detection Platform from Molecular Devices (Sunnyvale, CA, USA).

## Data Availability

The RNA-seq data associated with this manuscript have been submitted to Gene Expression Omnibus (GEO) and are available under the series accession GSE290668.

## Supplementary Materials

The following supporting information can be downloaded at: https://www.mdpi.com/article/10.3390/ijms26062442/s1.

## Abbreviations

The following abbreviations are used in this manuscript:

CDL	calcium dibutyroyllysinate
CTL	control
DEG	differentially expressed gene
FDR	false discovery rate
FGM	fibroblast growth media
MITF	microphthalmia-associated transcription factor
PC	principal component
LYS	lysine
PAD	peptidyl arginine deiminase
ZDL	zinc dibutyroyllysinate
