# Supplementary material for: A Novel Butyrate Derivative, Zinc Dibutyroyllysinate, Blunts Microphthalmia-Associated Transcription Factor Expression and Up-Regulates Retinol and Differentiation Pathway mRNAs in a Full-Thickness Human Skin Model
Source: Int J Mol Sci. 2025 Mar 9;26(6):2442. doi: 10.3390/ijms26062442 (PMC11942002; doi:10.3390/ijms26062442)
Supplement: Supplementary file 1 [file ijms-26-02442-s001.zip › ijms-3495455-supplementary.pdf]

Supplemental Material

A novel butyrate derivative, zinc dibutyroyllysinate, blunts MITF expression and up-regulates retinol and differentiation pathway mRNAs in a full thickness human skin model

William R. Swindell[1,*], Krzysztof Bojanowski[2], Geovani Quijas[3], Ratan K. Chaudhuri[4]

[1]University of Texas Southwestern Medical Center, Department of Internal Medicine.

[2]Sunny BioDiscovery Inc., Santa Paula, CA, USA.

[3]Sunny BioDiscovery Inc., Santa Paula, CA, USA.

[4]Sytheon (a Hallstar company), Boonton, NJ, USA.

[*]Corresponding Author: William.Swindell@UTSouthwestern.edu

Supplemental figure legends

Figure S1. Differential expression analyses. (A, E, I, M, Q) Principal component plots. Samples are plotted with respect to the first two principal component axes. Only the six samples involved in each comparison are shown. (B, F, J, N, R) Raw p-value distributions. (C, G, K, O, S) Volcano plots. The number of differentially expressed genes is shown at two significance thresholds (top margin; open circle: $P < 0.05$ with $FC > 1.5$ or $FC < 0.67$; asterisk: $FDR < 0.10$ with $FC > 1.5$ or $FC < 0.67$). (D, H, L, P, T) MA plots. The number of differentially expressed genes is again shown at two significance thresholds (same criteria as volcano plots).

Figure S2. Aging- and senescence-related genes. (A) Mean FC for aging-related genes listed in at least 3 of 5 database sources. (B) Mean FC of senescence-induction genes (CellAge database). (C) Mean FC of senescence-inhibition genes (CellAge database). In (A) - (C), gray regions outline the null distribution obtained by randomly sampling genes and calculating the mean FC for each sample. Red circles denote the average FC for each treatment (comparison to CTL group) and the average is also listed in the bottom margin (*$P < 0.05$, based on null distribution). (D) Aging-related genes (3+/5 database sources). (E) Senescence-inducing genes (CellAge database). (F) Senescence-inhibiting genes (CellAge database). In (D) - (F), FC is plotted for each gene ($P > 0.05$, circle; $P < 0.05$, asterisk). Genes were selected based on the value P_{min} = min(P1, P2, P3), where P1,…, P3 are p-values calculated for each of the 3 comparisons to the CTL treatment (LYS vs. CTL, CDL vs. CTL, ZDL vs. CTL). The top 30 genes with lowest value of P_{min} are shown in each figure.

Figure S3. Fold-change scatterplots. Each plot compares FC estimates associated with test compound response (horizontal axis) and the melanoma expression profile (vertical axis) (GSE7553). The proportion of genes associated with each quadrant is listed in the top margin (red font, $P < 0.05$, Pearson's chi-squared test) and represented graphically by the sidebar (right margin). The yellow ellipse outlines the middle 90% of genes based on the Mahalanobis distance. The yellow line denotes the least squares regression fit. The Spearman rank correlation and associated p-value are shown (bottom-left or bottom-right).

Figure S4. Read mapping results. (A) Read count (pre-filter). (B) Read count (post-filter). (C) Mapping rate. (D) Intragenic mapping rate. (E) Exonic mapping rate. (F) rRNA mapping rate.

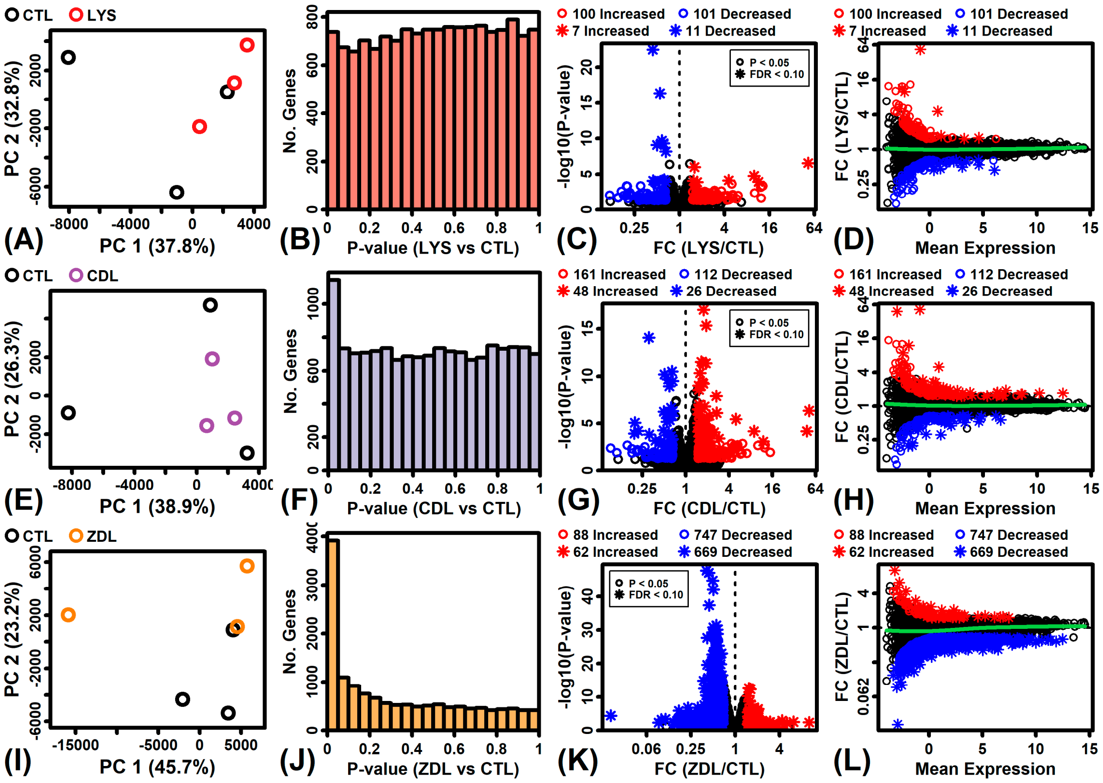

Figure S1. Differential expression analyses.

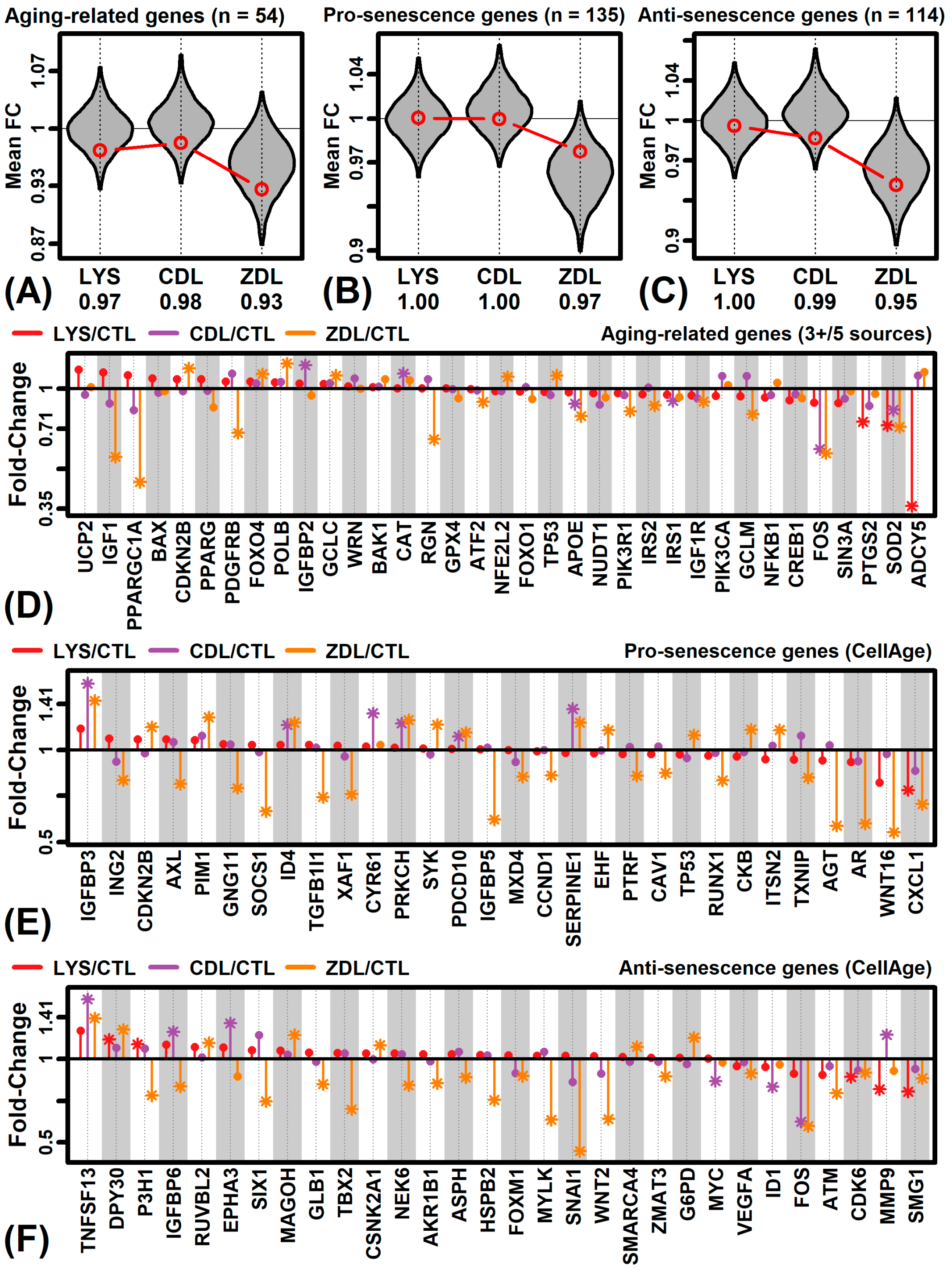

Figure S2. Aging- and senescence-related genes.

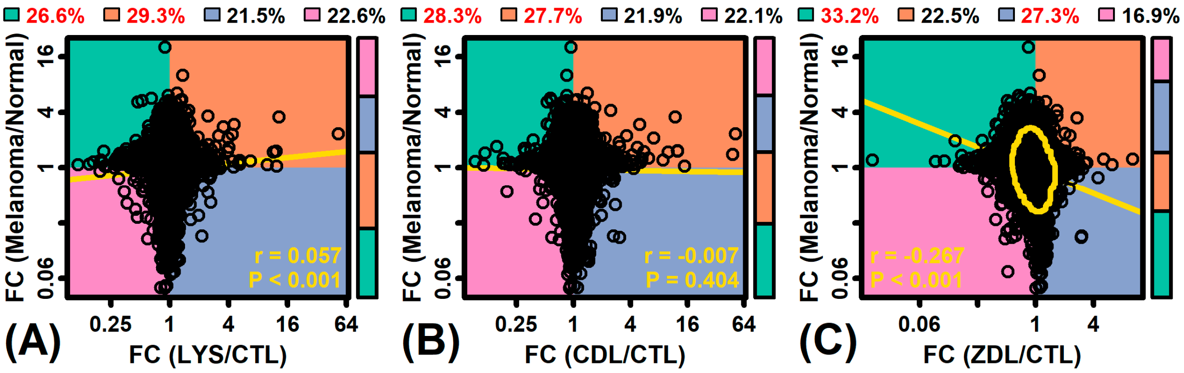

Figure S3. Fold-change scatterplots.

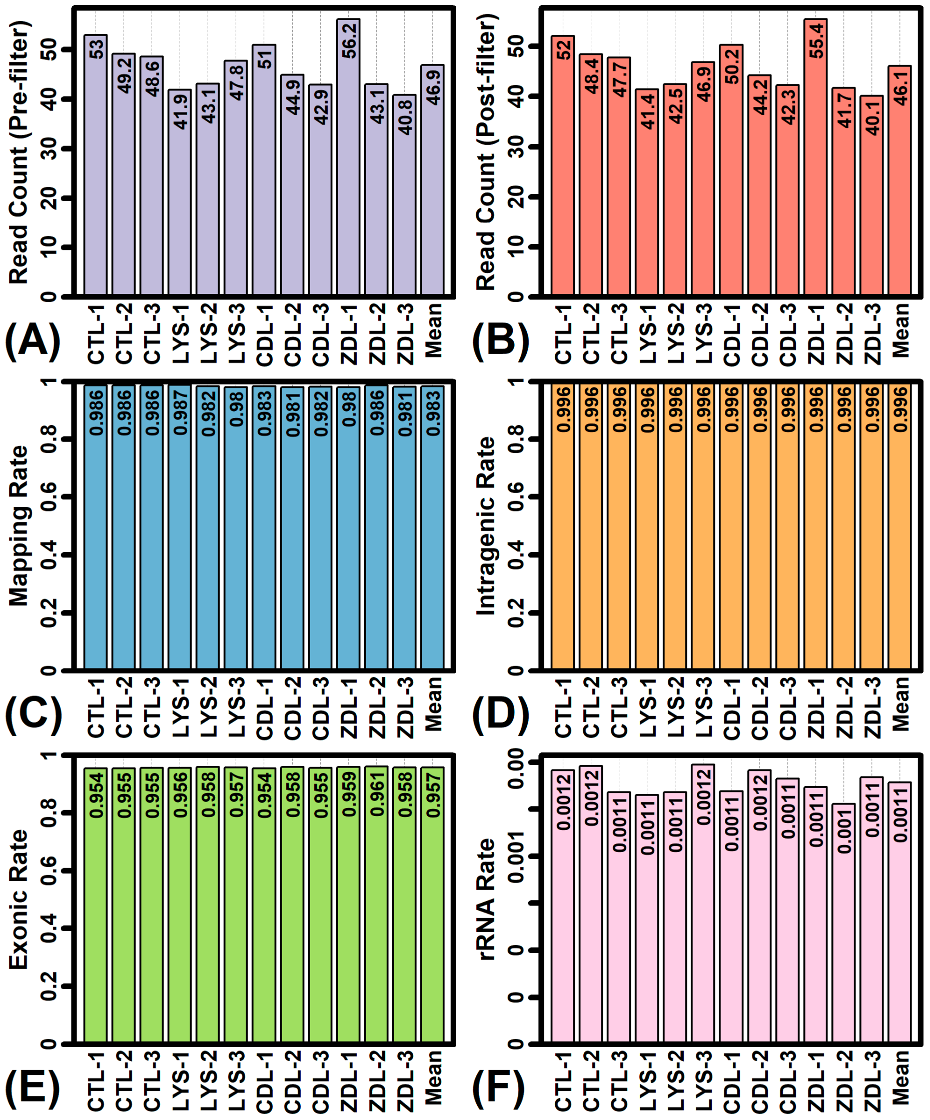

Figure S4. Read mapping results.
